# Association between Patient–Provider Communication and Self-Perceived Mental Health in US Adults with Cancer: Real-World Evidence through Medical Expenditure Panel Survey

**DOI:** 10.3390/diseases10040088

**Published:** 2022-10-15

**Authors:** Briana M. Choi, Mavis Obeng-Kusi, David R. Axon

**Affiliations:** Department of Pharmacy Practice & Science, R. Ken Coit College of Pharmacy, The University of Arizona, 1295 N Martin Ave, P.O. Box 210202, Tucson, AZ 85721, USA

**Keywords:** medical expenditure panel survey, mental health, cancer, patient–physician communication, patient–provider communication

## Abstract

Using real-world data, this retrospective cross-sectional study aimed to analyze the association between patient–physician communication and self-reported mental health from cancer patients using publicly available 2019 Medical Expenditure Panel Survey (MEPS) household component data. Four adjusted logistic regression models were conducted to analyze the association between patient–physician communication and self-perceived mental health among United States adult cancer patients, with each model assessing different aspects of patient–physician communication: being respected (respect), being listened to (listen), spending enough time (time), and being explained (explain). The main finding from this study was that only the time model showed a statistical significance, where patients who reported that their physician always spent enough time perceived their mental health as good. Other covariates that showed statistical significance with self-perceived mental health in cancer patients included age, income level, physical limitation, and limitation from pain. In conclusion, there were generally no statistically significant associations between patient–physician communication and mental health among cancer patients.

## 1. Introduction

In the United States (US), 9.5% of adults have been diagnosed with cancer, which is the second leading cause of death [1,2]. Cancer drugs account for 27% of the Food and Drug Administration’s (FDA’s) new drug approvals since 2010 [3]. In addition, 30 new drugs and biologic products have been approved for cancer treatments in 2020 alone [4]. Thus, there has been an increase in treatment options for patients and a corresponding increase in cancer survival rates [5]. However, increased treatment options and survival rates have not necessarily translated to decreased burdens for cancer patients. Despite the increased efforts to improve various aspects of health outcomes for cancer patients [5], patient burden remains substantial among these patients [6,7,8]. Additionally, in the current global coronavirus disease 2019 (COVID-19) pandemic, cancer patients’ stress and symptom burdens have exceeded previously reported levels [9]. As a result, many cancer patients suffer from mental health symptoms such as anxiety and depression [9].

One approach to help patients manage their mental health issues is good communication with their healthcare provider. Aspects of good patient–physician communication can include physicians showing respect to patients, physicians listening to patients, physicians spending enough time with patients, and physicians helping patients understand their conditions. The literature shows that there are several positive health outcomes from good patient–physician communications, as improved health outcomes are associated with effective patient–provider communication and patient satisfaction [10,11]. Increased participation of patients in preventative services such as cervical cancer, colorectal cancer, high cholesterol screenings and influenza vaccinations has occurred after effective patient–physician communications [12]. In oncology, this translated into increased numbers of patients participating in cancer screenings for breast, cervical, colon, and prostate cancer, including among underserved populations [12,13,14,15]. Besides increased utilization of preventative health services, patient–physician communication has resulted in several benefits, such as: increased probability of cancer-directed and stage-appropriate treatment for lung cancer [16]; higher perception of control over ovarian-related cancer symptoms [17]; and increased physical health and mental health quality of life for cancer survivors and prostate cancer patients [18,19].

Research has indicated that patients on medications for mental health conditions and their physicians both agreed that patient–physician communication and patient involvement is an important tool for their treatments and outcomes [20], which was also applicable to the underserved population with cancer [21]. In addition, a systematic review found those who had better patient–physician communication and relationships reported better adherence to their treatments [22]. However, little is known about the association between various aspects of patient–physician communication (such as physicians showing respect to patients, physicians listening to patients, physicians spending enough time with patients, and physicians helping patients understand their conditions) and mental health outcomes among patients with cancer in the US using real-world data. Real-world data, defined as data related to patient health status and/or care that are collected from a variety of sources, play a key role regarding how health and health care work in real-life settings [23]. There was one real-world data study that investigated the effect of patient–physician communication focusing on mental and emotional health, and it found that cancer survivors who had a conversation about emotional and social needs were at lower odds of having depression symptoms compared to those who did not [24]. Since we are interested in how certain aspects of patient–physician communication affect mental health on cancer patients, in this study we aimed to analyze the association between patient–physician communication and self-reported mental health status using real-world data.

## 2. Materials and Methods

### 2.1. MEPS and Study Design

The Medical Expenditure Panel Survey (MEPS) consists of a set of surveys for families and individuals, medical providers, and employers in the US, covering topics on cost and utilization of health care and health insurance. The MEPS, conducted by the Agency for Healthcare Research and Quality, utilizes a sample of the National Health Interview Survey and oversamples disabled and minority groups to collect data that represent the US population. There are two main components of the survey; the MEPS household component, which collects data from individual households (and is supplemented with data from medical providers), and the MEPS insurance component, which collects data from employers. The MEPS household component surveys are typically administered five times over a two-year timeline. In this cross-sectional retrospective database study, we used the 2019 MEPS full-year consolidated data file as this was the most recent available data at the time of the study. Participants of the survey provided oral informed consent for their voluntary participation [25].

### 2.2. Eligibility

Participants were included in the analyses if they were between 18 and 85 years of age, alive for the full calendar year, reported a diagnosis of cancer, and answered the self-perceived mental health question. Diagnosis of cancer was only asked to participants over 18 through a question: “Have you ever been told by a doctor or other health professional that you had cancer or a malignancy of any kind?”, with possible response options of “yes” or “no”.

### 2.3. Outcome Variable

The primary outcome was self-perceived mental health. Self-perceived mental health was assessed through a question: “In general, would you say that your mental health is excellent, very good, good, fair, or poor?” The response options for the item about mental health included “excellent”, “very good”, “good”, “fair”, and “poor” [26]. These were dichotomized into “excellent, very good, or good” (the good mental health group) and “fair or poor” (the poor mental health group) for this study. If the response was missing, then these data were not included in the analyses.

### 2.4. Independent Variable

Patient–physician communication was assessed using four variables: (1) doctor showed respect (respect model); (2) doctor listened to you (listen model); (3) doctor spent enough time with you (time model); and (4) doctor explained so you understood (explain model). The following items were used to determine the independent variables: (1) “How often a person’s doctors or other health providers showed respect for what the person had to say”, (2) “How often a person’s doctors or other health providers listened carefully to the person”, (3) “How often doctors or other health providers spent enough time with a person”, and (4) “How often a person’s doctors or other health providers explained things in a way the person could understand”. The response options for all items were “never”, “sometimes”, “usually”, and “always” [26].

### 2.5. Control Variables

Basic demographic information (sex, age, race, and ethnicity) was added to the four models to assess if there was a statistically significant difference between the good and poor mental health groups.

Based on the existing literature [27], additional potential factors that may affect mental health among patients with cancer were also added to the four models. These included education level (less than high school, completed high school, or some college education), marital status (married, single, or divorced/separated/widowed), employment (employed or unemployed), income level (poor, middle, or high income), physical limitation (yes or no), number of chronic conditions (less than five or five and above), and work limitation from pain (yes or no).

Number of chronic conditions were assessed from the following list: angina, arthritis, asthma, chronic bronchitis, coronary heart disease, diabetes, emphysema, hypercholesterolemia, hypertension, myocardial infarction, other unspecified heart disease, and stroke [26]. Cancer was not included as one of the comorbidities since all participants in the analyses had cancer.

We included work limitation from pain as its own variable instead of counting it as one of the chronic condition health conditions because there has been an association between pain and depression in cancer patients [28]. Work limitation from pain was identified from the question “During the past four weeks, pain interfered with normal work outside the home and housework”, thus it may not have been a chronic condition [26].

If responses for any of these variables were missing, then these data were not included in the analyses. Imputed or edited data were used for the analyses over raw data when both were provided [26].

### 2.6. Data Analysis

Chi-square tests were used to compare the patient characteristics per the mental health status. All statistical analyses, including the chi-square test and unadjusted and adjusted logistic regressions, were performed via SAS On Demand for Academics (SAS Institute Inc., Cary, NC, USA). Unadjusted and adjusted logistic regression models were each conducted for the four independent variables of interest (respect, listen, time, and explain models). Unadjusted logistic regression models were constructed first, followed by adjusted logistic regression models. Adjusted regression models included the covariates mentioned above. The analyses modeled good mental health, and poor mental health was the reference group. The a priori alpha level was 0.05. We accounted for the complex survey data using the PROC SURVEY commands in SAS and using the cluster and strata variables. Variance was calculated via the Taylor-series linearization technique. Weighting was used to produce nationally representative estimates. We assessed collinearity using the correlation technique and variance inflation factors, where a correlation greater than 0.8 or a variance inflation factor greater than five was deemed evidence of collinearity.

## 3. Results

### 3.1. Primary Outcomes

**Figure 1:**
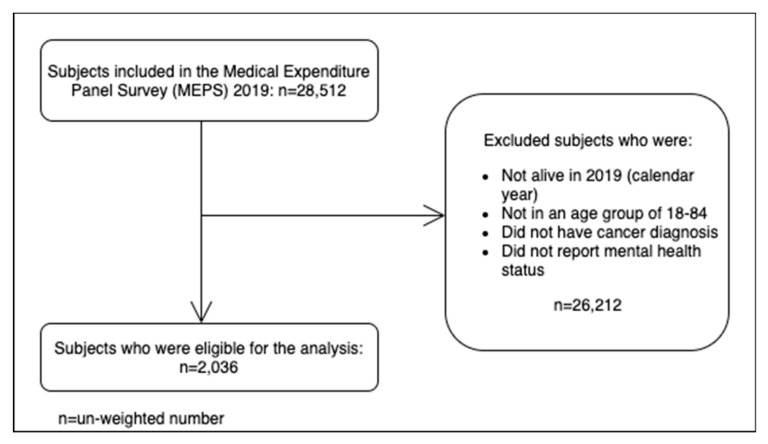
Subject selection flowchart.

Figure 1 shows the subject selection flowchart. A total of (unweighted) 2036 participants were included in the analyses after applying the inclusion criteria to the MEPS 2019 data (n = 28,512). There were 1800 participants (weighted percentage: 89.3%) who reported having a good self-perceived mental health while there were 236 participants (weighted percentage: 10.7%) who reported having poor mental health.

Table 1 summarizes the participant characteristics stratified by mental health groups (good vs. poor mental health). Information on sex, age, race, ethnicity, education, marital status, employment, income level, physical limitation, number of chronic conditions, and work limitation from pain were included. There were more females than males in both groups, although the good mental health groups had fewer females (54.8%) than the poor mental health group (63.4%). Both good and poor mental health groups had a greater prevalence of higher age groups. Majority of participants were White (90.1% for good mental health versus 84.1% for poor mental health). There were small portions of Black and other races for each group, although the good mental health group had lower percentages of Black (6.4% for good mental health versus 10.3% for poor mental health) and other races (3.6% for good mental health versus 5.6% for poor mental health). Most participants were non-Hispanic (94.0% for good mental health versus 90.1% for poor mental health). For education level, only 7.3% of persons who perceived good mental health received less than high school education, while 22.9% of those reporting poor mental health fell into this category. The proportion of individuals who completed high school (23.2% for good mental health versus 22.1% for poor mental health) or some college (69.5% for good mental health versus 55.1% for poor mental) in the good and poor mental health groups were different from each other. For marital status, the good mental group had a lower proportion of divorced/separated/widowed category by 10% (27.0% for good mental group versus 37.8% for poor mental health). The good mental health group had a lower proportion of unemployed participants compared to the poor mental health group (53.4% for good mental health versus 72.4% for poor mental health). The income level showed the opposite trend. Those who reported good mental health most commonly were in the high-income group (56.2%), while those with poor self-perceived mental health had the majority in the poor/near poor/low-income group (49.4%). While only 20.0% of participants with good self-perceived mental health had physical limitations, a little more than half of the poor mental health group had physical limitations (55.5%). For the number of chronic conditions, both groups had a majority in ≥5 chronic conditions (88.8% for good mental health versus 74.3% for poor mental health). Approximately half (51.7%) of those with good mental health reported a work limitation due to pain while a majority (81.7%) of those with poor self-perceived mental health reported the same. All variables were statistically significantly different between the good and poor mental health groups with the exception of ethnicity (*p* = 0.06) (Table 1).

### 3.2. Unadjusted Logistic Regression

Among the four unadjusted logistic regression (respect, listen, time, explain), only two models showed statistically significant odds ratios (ORs). In the unadjusted “respect” model, while participants who answered “always” being respected by the doctor had an OR of 6.3 (95% confidence interval (CI): 1.6–24.8), those who reported usually being respected by the doctor had an OR of 4.6 (1.2–17.9) compared to the reference group (“never”). Another model that showed statistical significance was the “time” model. Participants who reported the doctor always spending enough time with them had an OR of 4.6 (1.7–12.2) and those who reported the doctor usually spending enough time had an OR of 3.2 (1.2–8.6) compared to the “never” group (Table 2).

### 3.3. Adjusted Logistic Regression

In all four models, there was no significant association between the independent variable and good mental health status except for participants who reported their doctors as “always” having enough time for them (versus never having enough time), which showed a statistically significant association with good mental health (OR = 3.0, 95% CI = 1.1–8.4). However, we found that compared to age 64–84, age 18–44 and age 45–64 were associated with 0.1 times and 0.6 times, respectively, lower odds of reporting good mental health in all four models. Those classified as having a poor income level also had 0.4 times lower odds of reporting good self-perceived mental health compared to a high-income level in all four models. All four models also showed that participants without a physical limitation had 3.4 times higher odds of perceiving good mental health compared to those with a physical limitation, and participants without a work limitation from pain had approximately 1.8 times higher odds of reporting good mental health compared to those who did have a work limitation (Table 3).

## 4. Discussion

There were two key findings from this study. First, there was typically no association between patient–physician communication except for one level of the amount of time spent between patients and physicians and self-perceived mental health. Second, some of the covariates showed a significant association with perceived mental health status (age, income level, physical limitation, and work limitation from pain). These findings are discussed in detail below.

There was no statistically significant association between patient–provider communication and mental health status in all four adjusted logistic regression models, with the exception of one level of analysis in the time model. Participants who reported “always” spending enough time with the physician had three times higher odds of reporting good mental health than those who reported “never” spending enough time with the physician. However, in the unadjusted models, there were four instances of significant associations between patient–provider communication and good mental health status: “always” and “usually” feeling respected by the physician and “always” and “usually” spending enough time with the physician. However, the adjusted model results are more relevant since they account for the presence of covariates. This finding indicates that there is no association between patient–physician communication and mental health status except when the patient reported that the physician always spent enough time with them. Although this study examined four aspects of patient–physician communication, there may be other aspects of communication that need to be investigated to assess their associations with mental health to expand our understanding of this topic. Further investigation exploring how the amount of time spent with the patient affects patients’ mental health also should be conducted.

There is limited literature available on the association between the four aspects of patient–physician communication and mental health with which to compare our study findings, which demonstrates the value of our study to the literature. However, previous research has found associations between positive health outcomes and effective patient–physician communication in various other health conditions [10,11,12,13,14,15,16,17,18,19]. Therefore, it was interesting that the association between patient–physician communication and mental health status was found only in one of our models but not for the other three models. One previous study that used 2016–2017 MEPS data to analyze the relationship between patient–physician communication, specifically on emotional and social needs and mental health among cancer survivors, found that cancer survivors who discussed emotional and social needs had 0.45 times lower odds of having depressive symptoms [24]. The same cancer survivors showed 1.97 times higher odds of having a beneficial experience [24]. However, this study used the question: “at any time since you were first diagnosed with cancer, did any doctor or other healthcare provider, discuss your emotional or social needs related to your cancer, its treatment, or the lasting effects of that treatment?” instead of the four variables (respect, listen, time, and explain) used in our study, which may account for the different results [24]. This focused on communication about emotional and social needs, whereas we focused on general communication between cancer survivors and physicians, which may also account for some of the differences in our findings.

Because of some differences between previous research and our study, there may or may not be a critical role of patient–physician communication on patients’ mental health. However, the patient–physician communication was evaluated at a general level, meaning that the communication was not specifically between cancer patients and oncologists, but rather was between cancer patients and any type of physician. Moreover, this was a cross-sectional study, meaning that there was a lack of temporal causal relationship between patient–physician communication and self-perceived mental health. Therefore, since only spending enough time showed the statistical significance from our findings, future studies analyzing the role of patient–physician communication between cancer patients and oncologists, including various aspects of communication, and self-reported mental health are warranted to further investigate this association. Understanding which aspect of patient–physician communication impacts patients’ mental health status will not only help with improving cancer patients’ mental health outcomes but may also result in a better understanding of what physicians can do to help alleviate patients’ mental health symptoms, especially during the current situation of the COVID-19 pandemic. As more data become available, a future systematic review with a meta-analysis should be conducted to gain a greater understanding of the association between the patient–physician relationship and mental health status among US adult cancer patients. Additional studies using alternative methods, for example, qualitative research using interviews or focus groups, could also be conducted to capture a more humanistic assessment of this relationship.

Since there was a statistically significant association between patient–physician communication and self-perceived mental health status only in the time model, our findings suggest that only certain aspects of communication between patients and physicians may be an important consideration for US cancer patients. Other variables (covariates) that this study found to have a statistically significant association with good mental health status among US cancer patients are explained in the following paragraphs.

The first covariate was age. Younger cancer patients were less likely to have reported good mental health when compared to the older age group. Similar to our findings, Linden et al. found that among cancer patients in Canada, younger age was associated with higher rates of depression [29]. They also found that more than half of cancer patients younger than 50 years of age had either clinical or subclinical levels of anxiety. Linden et al. explained that this finding may be due to a higher level of disruption in daily life compared to older patients. Linden et al. used depression specifically as their outcome of interest, whereas in the current study we used mental health more broadly [13]. Our findings support those found by Linden at al. and suggest that younger patients with cancer may require extra support in terms of mental health assessment and management.

Another covariate that had a significant association with good mental health status was income level. Specifically, participants with poor/near poor/low income had lower odds of reporting good mental health than those with high income. This finding agrees with the findings of the study by Anuk et al. where low-income Turkish outpatient oncology patients seeking help with mental health disorders had higher odds of having mood disorders, adjustment disorders, and anxiety disorders [30].

Additionally, the current study found those who reported not having a physical limitation were at higher odds of reporting good mental health status. A similar finding was shown among community center cancer patients aged 20 years and older who were undergoing cancer treatment. Immobility in these patients was highly correlated to patients’ reported level of depression [31]. However, Given et al.’s study analyzed immobility specifically due to cancer and treatments, which was different from the general physical limitation included in our study. Nonetheless, both our analyses and Given et al.’s study showed that regardless of causes for physical limitation, limitation on physical function was positively correlated with poor mental health [31].

The final covariate that showed a significant association with perceived good mental health was work limitation from pain. Those who reported not having a work limitation due to pain were at higher odds of reporting good mental health compared to those who did. Given et al. found that symptoms that included pain were highly correlated to the level of depression in community center cancer patients [31]. Once again, Given et al. defined pain as those from cancer and treatments associated with cancer, whereas we defined pain as general pain not specifically related to cancer. Moreover, Given et al. combined pain with other symptoms such as insomnia, coughing, and changes in appetite, bowel movements, concentration, appearance, and general outlook, but we used pain as its own covariate in the model. Although our findings are similar to Given et al.’s findings in that pain was associated with lower odds of good mental health status among cancer patients, it is important to confirm our findings through future studies since pain in Given et al. was combined with other symptoms [31]. Once again, including the work limitation from the pain covariate, all these covariates that showed statistical significance in relation to mental health status should be confirmed with future studies.

A limitation of this study is the use of self-reported and secondary data instead of data collected for the purpose of this study. There is a potential recall bias from this type of data even though the surveys were conducted every 4–5 months. Another limitation includes lack of a temporal relationship between patient–physician communication and mental health in US adult cancer patients due to the cross-sectional nature of the study and lack of specific data on patient–physician communication between cancer patients and oncologists. Self-perceived mental health was measured using a general questionnaire item that may have been interpreted subjectively by respondents. Future research is needed to investigate the relationship between patient–physician communication and mental health status using a more targeted measure. Patient–physician communication was analyzed using four variables each with four levels of measurement (“never”, “sometimes”, “usually”, and “always”) available in the data set, however, future research should explore additional variables or definitions of communication. There is also a possibility of varied perspectives of patient–physician communications in cases where participants had more than one physician, which this study was unable to capture.

## 5. Conclusions

Among US adults with cancer diagnosis, age 18 to 85, there were generally no statistically significant relationships between patient–physician communication and self-perceived poor mental health except for spending enough time. However, other variables including age, income level, physical limitation, and work limitation from pain had statistically significant associations with poor mental health status in US cancer adults. Future research is warranted to investigate the relationship between patient–physician communication among cancer patients and oncologists and status using different approaches, and to further investigate the relationship between other factors and mental health.

Based on the results of our study, where there was not a statistically significant relationship between patient–physician communication and self-perceived mental health, the quality of patient–physician communication may not help with cancer patients’ mental health, while cancer patients spending enough time with physicians showed lower odds of poor mental health. Therefore, it is crucial to spend enough time with patients to ensure effective patient–physician communication. To further understand how effective communication between cancer patients and, specifically, oncologists affect mental health for the patients, future studies are warranted.

## Figures and Tables

**Table 1:** Population characteristics of United States (U.S.) adults with a diagnosis of cancer stratified by self-perceived mental health status.

Characteristics	Good Mental Health (Weighted n = 22,649,400) Weighted% (95% CI)	Poor Mental Health (Weighted n = 2,724,985) Weighted% (95% CI)	*p*-Value
Sex			0.02
Female	54.8 (52.3–57.3)	63.4 (56.4–70.5)	
Male	45.2 (42.7–47.7)	36.6 (29.5–43.6)	
Age			0.04
18–44	8.8 (7.1–10.6)	16.4 (7.7–25.0)	
45–64	37.4 (34.8–40.0)	35.1 (28.0–42.2)	
65–84	53.8 (50.9–56.8)	48.6 (40.8–56.3)	
Race			0.03
White	90.1 (88.4–91.7)	84.1 (78.8–89.4)	
Black	6.4 (4.9–7.8)	10.3 (5.6–15.0)	
Other *	3.6 (2.7–4.4)	5.6 (2.6–8.6)	
Ethnicity			0.06
Hispanic	6.0 (4.8–7.3)	9.9 (5.1–14.7)	
Not Hispanic	94.0 (92.7–95.2)	90.1 (85.3–94.9)	
Education			<0.01
Less than high school	7.3 (5.9–8.7)	22.9 (17.1–28.6)	
Completed high school	23.2 (20.7–25.8)	22.1 (16.4–27.8)	
Some college	69.5 (66.6–72.3)	55.1 (47.7–62.4)	
Marital Status			0.01
Married	63.6 (61.1–66.2)	50.2 (42.4–57.9)	
Single	9.3 (7.6–11.0)	12.0 (6.5–17.6)	
Divorced/separated/widowed	27.0 (24.6–29.5)	37.8 (31.0–44.5)	
Employment			0.01
Yes	46.6 (43.5–49.7)	27.6 (19.5–35.6)	
No	53.4 (50.3–56.5)	72.4 (64.4–80.5)	
Income level			<0.01
Poor/near poor/low	19.5 (17.4–21.6)	49.4 (40.9–57.9)	
Middle	24.3 (22.1–26.5)	23.5 (16.4–30.6)	
High	56.2 (53.1–59.3)	27.2 (20.4–34.0)	
Physical limitation			<0.01
Yes	20.0 (18.0–22.0)	55.5 (48.0–63.0)	
No	80.0 (78.0–82.0)	44.5 (37.0–52.0)	
Number of chronic conditions			<0.01
<5	11.2 (9.4–12.9)	25.7 (18.8–32.7)	
≥5	88.8 (87.1–90.6)	74.3 (67.3–81.2)	
Work limitation from pain			
Yes	51.7 (49.1–54.4)	81.7 (75.5–87.9)	
No	48.3 (45.6–50.9)	18.3 (12.1–24.5)	

n = number; CI: confidence interval; *: combined American Indian, Alaska native, Asian, Native Hawaiian, Pacific Islanders and multiple races into one category due to small number of group size in these categories; unweighted number of 2036 was included in the analyses with 236 in poor mental health and 1800 in good mental health. Chi-square tests were used across to assess differences in groups.

**Table 2:** Unadjusted odds ratios from the binary logistic regression for the four models (respect, listen, time, and explain) to assess the association between patient–physician communication and good mental health status.

Variables	OR (95% CI)
Respect	
Always	**6.3 (1.6–24.8)**
Usually	**4.6 (1.2–17.9)**
Sometimes	2.9 (0.7–12.4)
Never	Reference
Listen	
Always	3.8 (0.8–19.3)
Usually	2.8 (0.6–14.3)
Sometimes	3.8 (0.8–19.3)
Never	Reference
Time	
Always	**4.6 (1.7–12.2)**
Usually	**3.2 (1.2–8.6)**
Sometimes	1.6 (0.6–4.6)
Never	Reference
Explain	
Always	2.7 (0.8–8.7)
Usually	2.0 (0.6–6.4)
Sometimes	2.7 (0.8–8.7)
Never	Reference

OR: odds ratio; CI: confidence interval; **bolded** numbers indicate statistical significance; all the models were modeled with poor mental health status as the reference group.

**Table 3:** Adjusted odds ratios from the binary logistic regression for the four models (respect, listen, time, and explain) to assess the association between patient–physician communication and good mental health status.

Covariates	Model for Respect Adjusted OR (95% CI)	Model for Listen Adjusted OR (95% CI)	Model for Time Adjusted OR (95% CI)	Model for Explain Adjusted OR (95% CI)
Respect				
Always	2.2 (0.6–8.5)	-	-	-
Usually	1.8 (0.5–6.7)	-	-	-
Sometimes	2.7 (0.6–11.0)	-	-	-
Never	Reference	-	-	-
Listen				
Always	-	1.3 (0.2–9.4)	-	-
Usually	-	1.0 (0.1–7.1)	-	-
Sometimes	-	0.9 (0.1–6.6)	-	-
Never	-	Reference	-	-
Time				
Always	-	-	**3.0 (1.1–8.4)**	-
Usually	-	-	2.3 (0.8–6.3)	-
Sometimes	-	-	2.2 (0.7–6.6)	-
Never	-	-	Reference	-
Explain				
Always	-	-	-	3.2 (0.8–13.7)
Usually	-	-	-	2.3 (0.6–10.1)
Sometimes	-	-	-	1.1 (0.2–5.5)
Never	-	-	-	Reference
Sex				
Male	1.2 (0.8–1.7)	1.1 (0.7–1.7)	1.1 (0.7–1.7)	1.1 (0.7–1.7)
Female	Reference	Reference	Reference	Reference
Age				
18–44	**0.1 (0.1–0.3)**	**0.1 (0.1–0.4)**	**0.1 (0.1–0.4)**	**0.1 (0.1–0.4)**
45–64	**0.6 (0.4–0.9)**	**0.6 (0.4–0.9)**	**0.6 (0.4–0.9)**	**0.5 (0.4–0.8)**
65–84	Reference	Reference	Reference	Reference
Race				
White	1.2 (0.6–2.4)	1.2 (0.6–2.5)	1.2 (0.6–2.5)	1.1 (0.5–2.4)
Black	1.0 (0.4–2.6)	1.0 (0.4–2.5)	1.0 (0.4–2.7)	1.0 (0.4–2.5)
Other	Reference	Reference	Reference	Reference
Ethnicity				
Hispanic	0.5 (0.2–1.2)	0.5(0.2–1.2)	0.5 (0.2–1.2)	0.5 (0.2–1.2)
Not Hispanic	Reference	Reference	Reference	Reference
Education				
Less than high school	0.7 (0.4–1.1)	0.6 (0.4–1.1)	0.7 (0.4–1.1)	0.7 (0.4–1.3)
Completed high school	1.1 (0.7–1.7)	1.1 (0.7–1.7)	1.1 (0.7–1.7)	1.1 (0.7–1.7)
Some college	Reference	Reference	Reference	Reference
Marital Status				
Married	1.0 (0.7–1.5)	1.0 (0.7–1.5)	1.0 (0.7–1.5)	1.0 (0.7–1.5)
Single	1.6 (0.9–3.2)	1.7 (0.9–3.2)	1.7 (0.9–3.3)	1.6 (0.8–3.2)
Divorced/separated/widowed	Reference	Reference	Reference	Reference
Employment				
Yes	0.6 (0.3–1.0)	0.6 (0.3–1.0)	0.6 (0.3–1.0)	**0.5 (0.3–0.9)**
No	Reference	Reference	Reference	Reference
Income level				
Poor/near poor/low	**0.4 (0.2–0.6)**	**0.4 (0.2–0.6)**	**0.4 (0.2–0.6)**	**0.4 (0.2–0.6)**
Middle	0.7 (0.4–1.3)	0.7 (0.4–1.3)	0.7(0.4–1.2)	0.7 (0.4–1.2)
High	Reference	Reference	Reference	Reference
Physical limitation				
No	**3.4 (2.3–5.2)**	**3.4 (2.3–5.2)**	**3.4 (2.3–5.2)**	**3.4 (2.3–5.1)**
Yes	Reference	Reference	Reference	Reference
Number of chronic conditions				
<5	Reference	Reference	Reference	Reference
≥5	0.6 (0.4–1.1)	0.7 (0.4–1.1)	0.7 (0.4–1.1)	0.7 (0.4–1.1)
Work limitation from pain				
No	**1.8 (1.1–3.0)**	**1.8 (1.1–2.9)**	1.8 (1.1–2.9)	1.7 (1.1–2.8)
Yes	Reference	Reference	Reference	Reference

OR: odds ratio; CI: confidence interval: the four model variables (respect, listen, time, and respect) were not part of adjusted model for one another; these models were adjusted for sex, age, race, ethnicity, education, marital status, employment income, physical limitation, number of chronic conditions, and work limitation from pain. The adjusted logistic regression models had following Wald statistic of <0.0001 while c-statistics were: 0.804 for respect model, 0.803 for listen model, 0.806 for time model, and 0.808 for explain model. **Bolded** numbers indicate statistical significance with a priori alpha level of 0.05.

## Data Availability

The data presented in this study are available on request from the corresponding author.
